# Bis(1,3-dimethyl-1*H*-imidazolium) hexa­fluoro­silicate: the second monoclinic polymorph

**DOI:** 10.1107/S1600536813018242

**Published:** 2013-07-06

**Authors:** Chong Tian, Wanli Nie, Maxim V. Borzov

**Affiliations:** a College of Chemistry, Leshan Normal University, Binhe Rd 778, Leshan 614000, Sichuan Province, People’s Republic of China

## Abstract

The title compound, 2C_5_H_9_N_2_^+^·SiF_6_^2−^, (I), crystallized as a new polymorph, different from the previously reported one (I*a*) [Light *et al.* (2007 ▶) private communication (refcode: NIQFAV). CCDC, Cambridge, England]. The symmetry [space groups *P*2_1_/*n* for (I) and *C*2/*c* for(I*a*)] and crystal packing patterns are markedly different for this pair of polymorphs. In (I), all imidazolium cations in the lattice are nearly parallel to each other, whereas a herringbone arrangement can be found in (I*a*). In (I), each SiF_6_^2–^ dianion forms four short C—H⋯F contacts with adjacent C_5_H_9_N_2_^+^ cations, resulting in the formation of layers parallel to the *ac* plane. In (I*a*), the C—H⋯F contacts are generally longer and result in the formation of layers along the *bc* plane.

## Related literature

For the structure of the previously reported polymorph of (I) and its solvatomorph 6C_5_H_9_N_2_^+^·3SiF_6_^2−^·CH_3_OH, see: Light *et al.* (2007 ▶) and Tian *et al.* (2013 ▶), respectively. For an overview of polymorphism, see: Bernstein (2002 ▶); Linden (2011 ▶). For the practical importance of sterically non-hindered 1,3-dialkyl-1*H*-imidazolium salts with perfluoro anions of the main-group elements in the preparation of Arduengo carbene adducts, see: Tian *et al.* (2012 ▶). For graph-set notation, see: Etter *et al.* (1990 ▶); Bernstein *et al.* (1995 ▶); Grell *et al.* (1999 ▶).
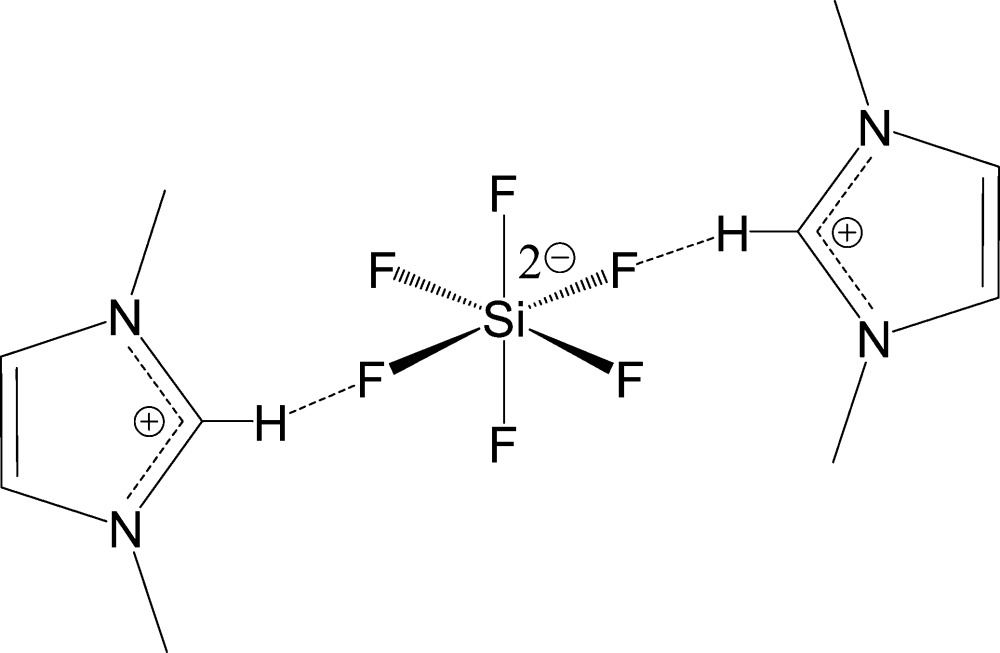


## Experimental

### 

#### Crystal data

2C_5_H_9_N^2+^·SiF_6_^2−^
*M* *_r_* = 336.38
Monoclinic, 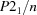

*a* = 8.2240 (8) Å
*b* = 9.7901 (9) Å
*c* = 8.7753 (9) Å
β = 90.106 (1)°
*V* = 706.53 (12) Å^3^
*Z* = 2
Mo *K*α radiation
μ = 0.23 mm^−1^
*T* = 296 K
0.40 × 0.39 × 0.05 mm

#### Data collection

Bruker SMART APEXII diffractometer
Absorption correction: multi-scan (*SADABS*; Sheldrick, 1996 ▶) *T*_min_ = 0.913, *T*_max_ = 0.988
3670 measured reflections
1373 independent reflections
1228 reflections with *I* > 2σ(*I*)
*R*_int_ = 0.020

#### Refinement

*R*[*F* ^2^ > 2σ(*F* ^2^)] = 0.028
*wR*(*F* ^2^) = 0.081
*S* = 1.09
1373 reflections
134 parameters
All H-atom parameters refined
Δρ_max_ = 0.18 e Å^−3^
Δρ_min_ = −0.22 e Å^−3^

### 

Data collection: *APEX2* (Bruker, 2007 ▶); cell refinement: *SAINT* (Bruker, 2007 ▶); data reduction: *SAINT*; program(s) used to solve structure: *SHELXS97* (Sheldrick, 2008 ▶); program(s) used to refine structure: *SHELXL97* (Sheldrick, 2008 ▶); molecular graphics: *SHELXTL* (Sheldrick, 2008 ▶) and *OLEX2* (Dolomanov *et al.*, 2009 ▶); software used to prepare material for publication: *SHELXTL* and *OLEX2*.

## Supplementary Material

Crystal structure: contains datablock(s) I, New_Global_Publ_Block. DOI: 10.1107/S1600536813018242/ld2107sup1.cif

Structure factors: contains datablock(s) I. DOI: 10.1107/S1600536813018242/ld2107Isup2.hkl

Supplementary material file. DOI: 10.1107/S1600536813018242/ld2107Isup3.cdx

Additional supplementary materials:  crystallographic information; 3D view; checkCIF report

## Figures and Tables

**Table 1:** Hydrogen-bond geometry (Å, °)

*D*—H⋯*A*	*D*—H	H⋯*A*	*D*⋯*A*	*D*—H⋯*A*
C1—H1⋯F3	0.884 (18)	2.164 (19)	2.9622 (17)	149.8 (14)
C2^i^—H2^i^⋯F2	0.934 (19)	2.26 (2)	3.1935 (18)	174.0 (5)

Symmetry code: (i) 
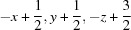
.

**Fig. 1.**
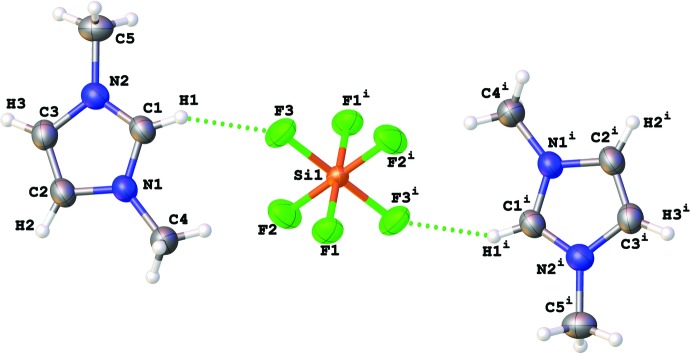
Formula unit of (I) along with atom labelling. Thermal ellipsoids are shown at 50% probability level. Symmetry code: (i) –x + 1, –y + 1, –z + 1. C—H···F contacts are depicted as dotted lines.

**Fig. 2.**
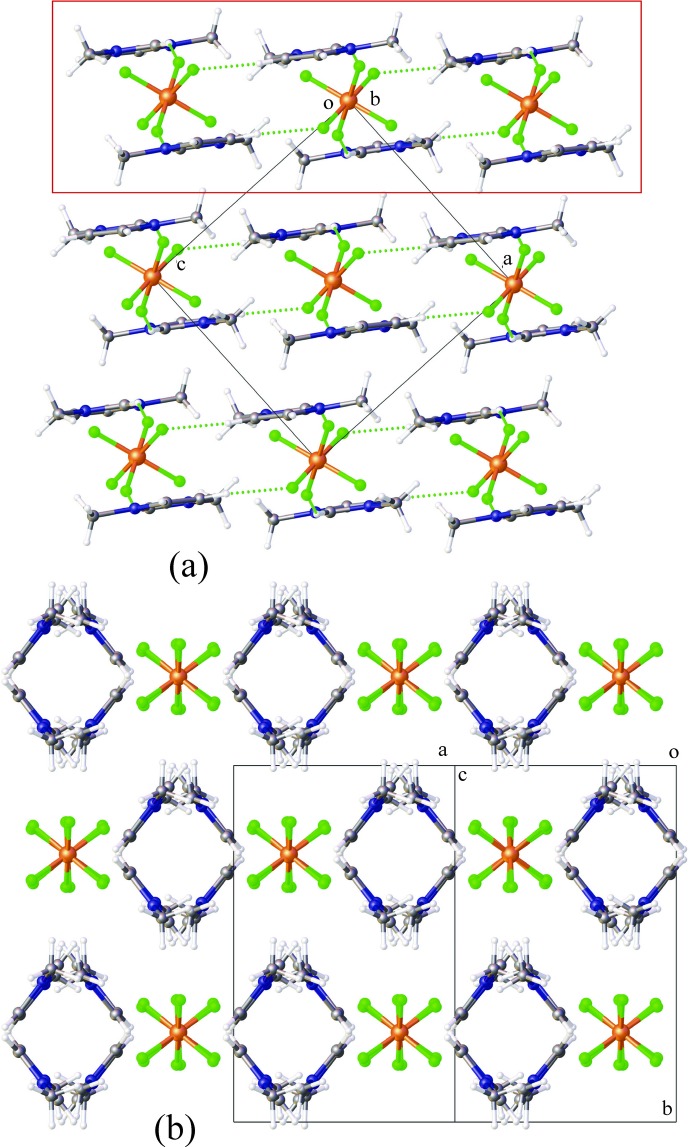
Packing diagrams for (I) [(a), a view along the b-axis] and (Ia) [(b), a view along the (–1, 0, 1) direction]. Ball-and-stick drawings. C—H···F contacts in (I) are depicted as dotted lines and omitted in (Ia) for clarity. One of the layers in the lattice of (I) is framed.

**Fig. 3.**
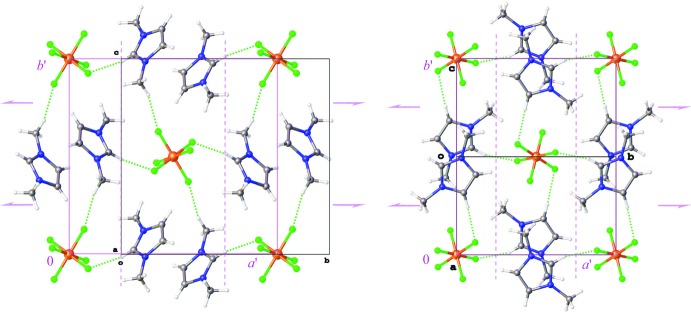
Ball-and-stick drawings of F···H binded layers (layer group p21/b11) in (Ia; left; a view along a-axis; N1=[D22(4)]H1'D22(4), N2=[C22(8)]H2,H1') and in (I) [right; a view along (1, 0, 1) direction; N1=[D22(4)]H4D22(4), N2=[C22(8)]H2,H4]. The F···H contacts are depicted as dotted lines. Symmetry diagrams for layer group p21/b11 are also provided in pink colour [a' = 11.988, b' = 11.258 Å for (Ia) and a' = 9.7901 (9), 12.0377 (9) Å for (I); the inversion centres are omitted for clarity]. For description of the graph set notation, see: Etter et al. (1990), Bernstein et al. (1995), Grell et al. (1999).

## supplementary crystallographic information

### Comment

Polymorphism of molecular crystals (including pseudopolymorphism and solvatomorphism) is an important object of structural studies [see a monograph (Bernstein, 2002) and an editorial paper (Linden, 2011)]. However, discovery of new polymorphic forms still remains a matter of serendipity.

Recently, being interested in preparation of a variety of sterically non-hindered 1,3-dialkyl-1*H*-imidazolium salts with main-group element perfluorato anions as potential precursors of Arduengo carbene adducts with the Group 13–15 element fluorides (Tian *et al.*, 2012), we analyzed materials obtained by re-crystallization of crude bis(1,3-dimethyl-1*H*-imidazolium) hexafluorosilicate, [C_5_H_9_N_2_^+^]_2_[SiF_6_^2–^], from either ethanol or methanol solutions. While crystallization from ethanol afforded only the solvent-free [C_5_H_9_N_2_^+^]_2_[SiF_6_^2–^], (I), the material obtained from methanol presented both (I) (crystals grow on the walls of a vessel above the solution surface during its gradual evaporation into air) and its adduct with methanol, {[C_5_H_9_N_2_^+^]_2_[SiF_6_^2–^]}_3_(CH_3_OH), (II), crystals of which grow at the bottom of a vessel under the layer of the mother liquor. In the latter case, single crystals of (I) and (II) could be easily separated manually. Identity of the single crystals of (I) prepared from EtOH and MeOH was proved by the unit cell measurements. Due to a better quality of the sample of (I) grown from ethanol, only these data are provided and will be referred to further in the discussion.

The β-angle very close to 90° presented a difficulty in determination of the actual crystal system and space group (*P*2_1_/*n*) for (I). The Si-atoms are positioned on inversion centres. Each [SiF_6_^2–^] moiety forms two pairs of noticeable F···H contacts: F3···H1 and F2···H2^ii^ [symmetry code: (ii) –*x* + 1/2, *y* + 1/2, –*z* + 3/2] and their centrosymmetric equivalents (see Table 1 and Fig. 1). In (I), the plane of the imidazolium ring is nearly perpendicular to the crystallographic [010] plane [interplanar angle 87.25 (4)°] that results in a nearly parallel arrangement of all imidazolium moieties in the lattice (see Fig. 2a). The F···H contacts connect the imidazolium cations and the [SiF_6_^2–^] dianions into layers parallel to the [101] plane (highlightened on Fig. 2a) consisting of the first- and second-order networks *N*_1_=*D*_2_^2^(4)*D*_2_^2^(4) and *N*_2_=*C*_2_^2^(8)]. Any interlayer C-H···F contacts shorter than 2.5 Å are absent in (I).

The previously reported polymorph (I*a*) (Light *et al.*, 2007) has space group *C*2/*c*. The principal geometrical parameters of ions in (I) and (Ia) are similar. However, the packing patterns in (I) and (I*a*) are distinctly different (compare Figs. 2a and 2 b). Overall, the packing in (I*a*) is less dense than that found in (I) [the respective calculated densities *D_x_* are 1.507 Mg m^-3^ for (I*a*) at 120 K and 1.581 Mg m^-3^ for (I) at 296 K. In (Ia), the H···F contacts are longer than in (I) and form the first- and second-order networks *N*_1_=*D*_2_^2^(4)*D*_2_^2^(4) and *N*_2_=*C*_2_^2^(8) seemingly similar to those in (I) (primary and secondary contact lenghts are equal to 2.334 and 2.359 Å, respectively). This network similarity, however, is only apparent because the secondary H···F contacts in (I*a*) are formed not by H^4^, but by a H^1'^-atom of the Me-group (superscripts here denote the positions in the imidazolium moiety). As recommended previously by Etter *et al.* (1990), the graph set descriptors for (I) and (I*a*) could be augmented, respectively, as *N*_1_=[*D*_2_^2^(4)]_H4_*D*_2_^2^(4), *N*_2_=[*C*_2_^2^(8)]_H2,H4_ and *N*_1_=[*D*_2_^2^(4)]_H1'_*D*_2_^2^(4), *N*_2_=[*C*_2_^2^(8)]_H2,H1'_ that allows to exclude any ambiguity.

The longer contacts in (I*a*) also form non-interconnected layers in its crystal lattice. The neighbour pairs of layers in (I*a*) are connected by the *C*-centering translation, *C*_2_ rotation, *n*-glide reflection, and inversion operations. Moreover, these layers belong to the same layer group as it is observed in (I; *p*2_1_/*b*11), with its *a*' and *b*' parameters being comparable [9.7901 (9) and 12.0377 (9) Å for (I) and 11.988 and 11.258 Å for (I*a*); see Fig. 3; priming is used to distinguish between unit cell axes *a* and *b* and layer-related axes *a*' and *b*']. Distances between adjacent inversion-related pairs of imidazolium rings are also close (the interplane distances are 3.418 (2) and 3.449 Å in (I) and (I*a*), respectively]. Fig. 3 also illustrates that the layers within (I) and (I*a*) can be converted one into another by a diffusionless transformation which can be best described as continuous rotations of the inversion-related pairs of imidazolium cations and SiF_6_^2–^ groups around the corresponding centers accompanied with dilations/contractions of the layers along the *a*' and *b*' directions. Transformation of the entire lattice of (I*a*) into that of (I) also requires a mutual (0, 1/2, 0) shear of adjacent layers in the *b*-direction what results in vanishing of centering translations and conversion of *C*_2_ rotations into 2_1_ screw ones. Being a continuous transformation, such a layer shift, however, can not be classified as a "diffusionless" one. Thus, any direct first-order phase transition between (I) and (I*a*) is hardly believable. Unfortunately, the lack of the information about the sample crystal of (I*a*) [in the corresponding Private communication to the CCDC (Light *et al.*, 2007), no data on the crystallization conditions are provided] does not allow us to outline the actual reasons of the (I)/(I*a*) polymorphism.

The structure of solvated crystals {[C_5_H_9_N_2_^+^]_2_[SiF_6_^2–^]}_3_(CH_3_OH), (II) is reported separately (Tian *et al.*, 2013).

### Experimental

Crude 1,3-dimethyl-1*H*-imidazolium hexafluorosilicate was prepared by a reaction of 1,3-dimethyl-1*H*-imidazolium iodide and disilver hexafluorosilicate (molar ratio 2:1) in distilled water. Concentration of the filtrate till dryness followed by re-crystallization from ethanol gave (I) in an almost quantitative yield. If methanol is used as a solvent, crystals of both (I) and (II) are formed. Single crystals of (I) suitable for X-ray diffraction analysis were picked up directly from the material (when methanol was used as a solvent, the crystals located on the vessel walls above the solution surface were selected). Identity of the single crystals of (I) grown from EtOH and MeOH was proved by unit cell measurements. Melting point measurements were performed with a Microscopic Melting Point X4 apparatus (Beijing MAISIQI High-Tech Co., Ltd.)

### Refinement

All non-H atoms were refined anisotropically. All H-atoms were found from the difference Fourier synthesis and refined isotropically.

### Crystal data

**Table:**

2C_5_H_9_N^2+^·SiF_6_^2^^−^	*F*(000) = 348
*M**_r_* = 336.38	*D*_x_ = 1.581 Mg m^−^^3^
Monoclinic, *P*2_1_/*n*	Melting point: 550 K
Hall symbol: -P 2yn	Mo *K*α radiation, λ = 0.71073 Å
*a* = 8.2240 (8) Å	Cell parameters from 3479 reflections
*b* = 9.7901 (9) Å	θ = 2.3–28.3°
*c* = 8.7753 (9) Å	µ = 0.23 mm^−^^1^
β = 90.106 (1)°	*T* = 296 K
*V* = 706.53 (12) Å^3^	Plate, colourless
*Z* = 2	0.40 × 0.39 × 0.05 mm

### Data collection

**Table:**

Bruker SMART APEXII diffractometer	1373 independent reflections
Radiation source: fine-focus sealed tube	1228 reflections with *I* > 2σ(*I*)
Graphite monochromator	*R*_int_ = 0.020
Detector resolution: 8.333 pixels mm^-1^	θ_max_ = 26.0°, θ_min_ = 3.2°
phi and ω scans	*h* = −10→10
Absorption correction: multi-scan (*SADABS*; Sheldrick, 1996)	*k* = −12→9
*T*_min_ = 0.913, *T*_max_ = 0.988	*l* = −10→8
3670 measured reflections	

### Refinement

**Table:**

Refinement on *F*^2^	Secondary atom site location: difference Fourier map
Least-squares matrix: full	Hydrogen site location: difference Fourier map
*R*[*F*^2^ > 2σ(*F*^2^)] = 0.028	All H-atom parameters refined
*wR*(*F*^2^) = 0.081	*w* = 1/[σ^2^(*F*_o_^2^) + (0.0471*P*)^2^ + 0.1056*P*] where *P* = (*F*_o_^2^ + 2*F*_c_^2^)/3
*S* = 1.09	(Δ/σ)_max_ < 0.001
1373 reflections	Δρ_max_ = 0.18 e Å^−^^3^
134 parameters	Δρ_min_ = −0.22 e Å^−^^3^
0 restraints	Extinction correction: *SHELXL*, Fc^*^=kFc[1+0.001xFc^2^λ^3^/sin(2θ)]^-1/4^
Primary atom site location: structure-invariant direct methods	Extinction coefficient: 0.065 (7)

### Special details

**Table:**

Experimental. A very tight closeness of the β-angle to 90° presented a certain difficulty in determination of the actual crystal system and the space group for (I).
Geometry. All e.s.d.'s (except the e.s.d. in the dihedral angle between two l.s. planes) are estimated using the full covariance matrix. The cell e.s.d.'s are taken into account individually in the estimation of e.s.d.'s in distances, angles and torsion angles; correlations between e.s.d.'s in cell parameters are only used when they are defined by crystal symmetry. An approximate (isotropic) treatment of cell e.s.d.'s is used for estimating e.s.d.'s involving l.s. planes.
Refinement. Refinement of *F*^2^ against ALL reflections. The weighted *R*-factor *wR* and goodness of fit *S* are based on *F*^2^, conventional *R*-factors *R* are based on *F*, with *F* set to zero for negative *F*^2^. The threshold expression of *F*^2^ > σ(*F*^2^) is used only for calculating *R*-factors(gt) *etc*. and is not relevant to the choice of reflections for refinement. *R*-factors based on *F*^2^ are statistically about twice as large as those based on *F*, and *R*- factors based on ALL data will be even larger.

### Fractional atomic coordinates and isotropic or equivalent isotropic displacement parameters (Å^2^)

**Table:**

	*x*	*y*	*z*	*U*_iso_*/*U*_eq_	
Si1	0.5000	0.5000	0.5000	0.02918 (18)	
F1	0.31022 (9)	0.53884 (9)	0.55758 (10)	0.0445 (3)	
F2	0.51424 (12)	0.38309 (9)	0.63991 (10)	0.0563 (3)	
F3	0.42533 (10)	0.38065 (9)	0.38175 (11)	0.0532 (3)	
N1	0.22279 (13)	0.08942 (11)	0.52963 (12)	0.0362 (3)	
N2	0.34964 (14)	−0.03111 (12)	0.36255 (13)	0.0373 (3)	
C1	0.31135 (17)	0.09435 (15)	0.40423 (16)	0.0387 (3)	
C2	0.20313 (19)	−0.04525 (15)	0.56970 (17)	0.0419 (3)	
C3	0.28241 (18)	−0.12049 (16)	0.46592 (16)	0.0423 (3)	
C4	0.1593 (2)	0.20729 (16)	0.61283 (19)	0.0462 (4)	
C5	0.4404 (2)	−0.06783 (19)	0.22545 (18)	0.0482 (4)	
H1	0.341 (2)	0.1721 (19)	0.3610 (19)	0.048 (4)*	
H2	0.146 (2)	−0.0695 (18)	0.658 (2)	0.054 (5)*	
H3	0.297 (2)	−0.2124 (19)	0.4561 (19)	0.050 (5)*	
H4A	0.218 (3)	0.282 (2)	0.590 (2)	0.074 (6)*	
H4B	0.048 (3)	0.227 (2)	0.580 (3)	0.080 (6)*	
H4C	0.162 (3)	0.188 (2)	0.718 (3)	0.093 (7)*	
H5A	0.538 (3)	−0.015 (2)	0.227 (3)	0.071 (6)*	
H5B	0.377 (3)	−0.058 (2)	0.141 (3)	0.074 (6)*	
H5C	0.479 (3)	−0.164 (3)	0.229 (3)	0.094 (7)*	

### Atomic displacement parameters (Å^2^)

**Table:**

	*U*^11^	*U*^22^	*U*^33^	*U*^12^	*U*^13^	*U*^23^
Si1	0.0312 (3)	0.0258 (3)	0.0306 (3)	−0.00187 (16)	0.00740 (19)	−0.00332 (17)
F1	0.0342 (4)	0.0494 (5)	0.0499 (5)	0.0012 (3)	0.0113 (4)	−0.0102 (4)
F2	0.0701 (6)	0.0494 (5)	0.0494 (5)	0.0135 (4)	0.0180 (4)	0.0160 (4)
F3	0.0480 (5)	0.0516 (5)	0.0599 (6)	−0.0113 (4)	0.0085 (4)	−0.0269 (4)
N1	0.0388 (6)	0.0355 (6)	0.0343 (6)	−0.0044 (4)	0.0013 (5)	0.0033 (4)
N2	0.0358 (6)	0.0419 (6)	0.0343 (6)	−0.0026 (5)	−0.0012 (5)	0.0007 (4)
C1	0.0409 (7)	0.0381 (7)	0.0371 (7)	−0.0041 (6)	0.0022 (6)	0.0054 (6)
C2	0.0483 (8)	0.0387 (7)	0.0386 (7)	−0.0075 (6)	0.0025 (6)	0.0060 (6)
C3	0.0488 (8)	0.0361 (7)	0.0419 (8)	−0.0048 (6)	−0.0018 (6)	0.0034 (6)
C4	0.0532 (9)	0.0391 (8)	0.0463 (9)	−0.0031 (7)	0.0107 (7)	−0.0017 (6)
C5	0.0454 (8)	0.0607 (10)	0.0384 (8)	0.0026 (7)	0.0036 (6)	−0.0050 (7)

### Geometric parameters (Å, º)

**Table:**

Si1—F3^i^	1.6783 (8)	N2—C5	1.4619 (19)
Si1—F3	1.6783 (8)	C1—H1	0.884 (19)
Si1—F2	1.6824 (8)	C2—C3	1.341 (2)
Si1—F2^i^	1.6824 (8)	C2—H2	0.934 (19)
Si1—F1	1.6849 (8)	C3—H3	0.912 (18)
Si1—F1^i^	1.6849 (8)	C4—H4A	0.90 (2)
N1—C1	1.3216 (18)	C4—H4B	0.98 (2)
N1—C2	1.3742 (18)	C4—H4C	0.94 (3)
N1—C4	1.4625 (19)	C5—H5A	0.96 (3)
N2—C1	1.3199 (18)	C5—H5B	0.91 (2)
N2—C3	1.3770 (19)	C5—H5C	0.99 (3)
			
F3^i^—Si1—F3	180.0	N2—C1—N1	109.20 (12)
F3^i^—Si1—F2	89.86 (5)	N2—C1—H1	128.1 (11)
F3—Si1—F2	90.14 (5)	N1—C1—H1	122.7 (11)
F3^i^—Si1—F2^i^	90.14 (5)	C3—C2—N1	107.19 (13)
F3—Si1—F2^i^	89.86 (5)	C3—C2—H2	131.8 (11)
F2—Si1—F2^i^	180.0	N1—C2—H2	121.0 (11)
F3^i^—Si1—F1	89.75 (4)	C2—C3—N2	107.14 (13)
F3—Si1—F1	90.25 (4)	C2—C3—H3	132.2 (11)
F2—Si1—F1	89.88 (4)	N2—C3—H3	120.6 (11)
F2^i^—Si1—F1	90.12 (4)	N1—C4—H4A	109.4 (14)
F3^i^—Si1—F1^i^	90.25 (4)	N1—C4—H4B	110.0 (13)
F3—Si1—F1^i^	89.75 (4)	H4A—C4—H4B	106.4 (18)
F2—Si1—F1^i^	90.12 (4)	N1—C4—H4C	108.6 (15)
F2^i^—Si1—F1^i^	89.88 (4)	H4A—C4—H4C	112 (2)
F1—Si1—F1^i^	180.00 (6)	H4B—C4—H4C	110 (2)
C1—N1—C2	108.26 (12)	N2—C5—H5A	106.3 (14)
C1—N1—C4	125.78 (12)	N2—C5—H5B	110.8 (14)
C2—N1—C4	125.94 (12)	H5A—C5—H5B	115.6 (19)
C1—N2—C3	108.21 (12)	N2—C5—H5C	111.8 (14)
C1—N2—C5	125.41 (13)	H5A—C5—H5C	104.5 (19)
C3—N2—C5	126.31 (13)	H5B—C5—H5C	107.7 (19)
			
C3—N2—C1—N1	0.05 (16)	C4—N1—C2—C3	−178.71 (14)
C5—N2—C1—N1	177.05 (13)	N1—C2—C3—N2	−0.22 (17)
C2—N1—C1—N2	−0.19 (16)	C1—N2—C3—C2	0.11 (17)
C4—N1—C1—N2	178.78 (13)	C5—N2—C3—C2	−176.85 (14)
C1—N1—C2—C3	0.26 (16)		

Symmetry code: (i) −*x*+1, −*y*+1, −*z*+1.

### Hydrogen-bond geometry (Å, º)

**Table:**

*D*—H···*A*	*D*—H	H···*A*	*D*···*A*	*D*—H···*A*
C1—H1···F3	0.884 (18)	2.164 (19)	2.9622 (17)	149.8 (14)
C2^ii^—H2^ii^···F2	0.934 (19)	2.26 (2)	3.1935 (18)	174.0 (5)

Symmetry code: (ii) −*x*+1/2, *y*+1/2, −*z*+3/2.
